# Inhibiting Human Parainfluenza Virus Infection by Preactivating the Cell Entry Mechanism

**DOI:** 10.1128/mBio.02900-18

**Published:** 2019-02-19

**Authors:** S. F. Bottom-Tanzer, K. Rybkina, J. N. Bell, C. A. Alabi, C. Mathieu, M. Lu, S. Biswas, M. Vasquez, M. Porotto, J. A. Melero, V. Más, A. Moscona

**Affiliations:** a Department of Pediatrics, Columbia University Medical Center, New York, New York, USA; b Center for Host-Pathogen Interaction, Columbia University Medical Center, New York, New York, USA; c Robert Frederick Smith School of Chemical and Biomolecular Engineering, Cornell University, Ithaca, New York, USA; d CIRI, International Center for Infectiology Research, Immunobiology of Viral Infections Team, INSERM U1111, University Claude Bernard Lyon 1, CNRS, UMR5308, Ecole Normale Supérieure de Lyon, Lyon, France; e Department of Microbiology, Biochemistry and Molecular Genetics, Rutgers Biomedical and Health Sciences, Public Health Research Institute, Newark, New Jersey, USA; f Centro Nacional de Microbiología and CIBER de Enfermedades Respiratorias, Instituto de Salud Carlos III, Madrid, Spain; g Department of Experimental Medicine, University of Campania Luigi Vanvitelli, Naples, Italy; h Department of Microbiology & Immunology, Columbia University Medical Center, New York, New York, USA; i Department of Physiology & Cellular Biophysics, Columbia University Medical Center, New York, New York, USA; St. Jude Children's Research Hospital; Johns Hopkins School of Public Health; Children's Hospital of Pittsburgh and University of Pittsburgh Medical Center

**Keywords:** antiviral, conformational antibody, fusion activation, paramyxovirus, viral fusion, viral glycoprotein antibody

## Abstract

Paramyxoviruses, specifically, the childhood pathogen human parainfluenza virus type 3, are internalized into host cells following fusion between the viral and target cell membranes. The receptor binding protein, hemagglutinin (HA)-neuraminidase (HN), and the fusion protein (F) facilitate viral fusion and entry into the cell through a coordinated process involving HN activation by receptor binding, which triggers conformational changes in the F protein to activate it to reach its fusion-competent state. Interfering with this process through premature activation of the F protein has been shown to be an effective antiviral strategy *in vitro.* Conformational changes in the F protein leading to adoption of the postfusion form of the protein—prior to receptor engagement of HN at the host cell membrane—render the virus noninfectious. We previously identified a small compound (CSC11) that implements this antiviral strategy through an interaction with HN, causing HN to activate F in an untimely process. To assess the functionality of such compounds, it is necessary to verify that the postfusion state of F has been achieved. As demonstrated by Melero and colleagues, soluble forms of the recombinant postfusion pneumovirus F proteins and of their six helix bundle (6HB) motifs can be used to generate postfusion-specific antibodies. We produced novel anti-HPIV3 F conformation-specific antibodies that can be used to assess the functionality of compounds designed to induce F activation. In this study, using systematic chemical modifications of CSC11, we synthesized a more potent derivative of this compound, CM9. Much like CSC11, CM9 causes premature triggering of the F protein through an interaction with HN prior to receptor engagement, thereby preventing fusion and subsequent infection. In addition to validating the potency of CM9 using plaque reduction, fusion inhibition, and binding avidity assays, we confirmed the transition to a postfusion conformation of F in the presence of CM9 using our novel anti-HPIV3 conformation-specific antibodies. We present both CM9 and these newly characterized postfusion antibodies as novel tools to explore and develop antiviral approaches. In turn, these advances in both our molecular toolset and our understanding of HN-F interaction will support development of more-effective antivirals. Combining the findings described here with our recently described physiologically relevant *ex vivo* system, we have the potential to inform the development of therapeutics to block viral infection.

## Abstract (precis)

Paramyxoviruses, including human parainfluenza virus type 3, are internalized into host cells by fusion between viral and target cell membranes. The receptor binding protein, hemagglutinin-neuraminidase (HN), and the fusion protein (F) facilitate viral fusion and entry into cells through a process involving HN activation by receptor binding, which triggers conformational changes in F to activate it to reach its fusion-competent state. Interfering with this process through premature activation of the F protein may be an effective antiviral strategy *in vitro*. We identified and optimized small compounds that implement this antiviral strategy through an interaction with HN, causing HN to activate F in an untimely fashion. To address that mechanism, we produced novel anti-HPIV3 F conformation-specific antibodies that can be used to assess the functionality of compounds designed to induce F activation. Both the novel antiviral compounds that we present and these newly characterized postfusion antibodies are novel tools for the exploration and development of antiviral approaches.

## INTRODUCTION

Acute respiratory infection is the leading cause of child mortality worldwide (1, 2). More than 20% of all acute lower respiratory infections are associated with paramyxovirus infection, and greater than 14% result in death (2). Paramyxoviruses and pneumoviruses account for the majority of childhood croup, bronchiolitis, and pneumonia cases (3), with human parainfluenza virus 3 (HPIV3) infections alone resulting in 11% of childhood respiratory hospitalizations in the United States (3, 4). There are currently no vaccines or antiviral therapies for parainfluenza viruses.

Paramyxovirus entry, including HPIV3 entry, is mediated by fusion of the viral and target host cell membranes at the cell surface. Virus-cell fusion results from coordinated action of the two envelope glycoproteins that comprise the viral entry machinery—a receptor binding protein, hemagglutinin neuraminidase (HN), and a fusion protein (F). Upon binding to sialic acid-containing target receptors, HN, a molecule with both receptor binding and cleaving activities, triggers and activates the F protein (5). Once F is activated, the hydrophobic fusion peptide inserts into the target host membrane and undergoes a series of structural rearrangements leading to association between heptad repeats (HR) at the C terminus and N terminus of the molecule (HRC and HRN, respectively) and subsequent fusion between the viral and cellular membranes (6).

The process of viral fusion and the extent to which it occurs are mediated by the various functions of HN and F. HN moderates receptor binding and cleavage, as well as stabilization and activation of the F protein. HN, a type II transmembrane protein, gives rise to these functions via coordination between its cytoplasmic domain, membrane-spanning region, stalk region, and a globular head, which contains the primary sialic acid binding site and neuraminidase active site, as well as a second sialic acid binding site that modulates activation of F. F influences the extent of fusion through its prefusion stability, kinetics of activation, and precursor cleavability. Fusion is moderated through a balance of these functions, with timing also playing an essential role.

Activation and the subsequent conformational change of the F protein must occur when F is in contact with the target cell membrane. If F assumes its elongated intermediate state or its postfusion state prematurely, the virus is rendered noninfectious. This sequence of events presents a possible antiviral target (3). We sought to identify molecules that irreversibly inactivate the viral fusion complex by stimulating HN to trigger F prior to receptor engagement. In a computational screen for small molecules that bind to the globular head domain of HN, we identified a compound that inhibited entry of a laboratory-adapted strain of HPIV3 into target monolayer cells and prevented infection. This compound interacts with HN prior to receptor engagement, thereby causing premature F activation and precluding viral entry (7). The prototype molecule (“CSC11”) interacts with HN without altering receptor binding avidity or receptor cleaving (neuraminidase) activity. The compound showed virucidal activity and an antiviral effect on laboratory-adapted HPIV3 in both cultured monolayer cells and human airway epithelium (HAE) cells (7).

The prototype compound provided proof of concept for the strategy of inducing HPIV to self-inactivate and points to the possibility of developing a new class of antivirals (7–10). We carried out systematic chemical modifications of each structural group in the CSC11 compound and assessed the efficacy of the derived compounds on the infectivity and other key properties of clinical isolate (field strain) viruses in authentic host tissues. We have also assessed the functions of receptor binding, receptor cleaving, and fusion as well as viral entry in the presence of these compounds and identified a modification that improves the antiviral effect (activity) without modulating receptor binding or cleaving activity. To further assess the functionality of these compounds *in vitro*, it is necessary to verify that the postfusion state of F has been achieved. As demonstrated previously by Melero and colleagues (11, 12), genetically engineered recombinant postfusion F proteins can be used to generate postfusion antibodies (Abs). To assess the functionality and mechanism of action of these compounds, we produced novel anti-HPIV3 postfusion antibodies (11, 12). The results presented here demonstrate the ability to make use of F protein pretriggering as an antiviral strategy and as a way to engineer a more potent antiviral. Here, we identified the mechanism of action of this new class of antiviral compound by analyzing the effect of these molecules on the functions and structures of HPIV3 surface glycoproteins.

## RESULTS

### Effect of chemical modification of prototype compound on antiviral activity.

**FIG 1:**
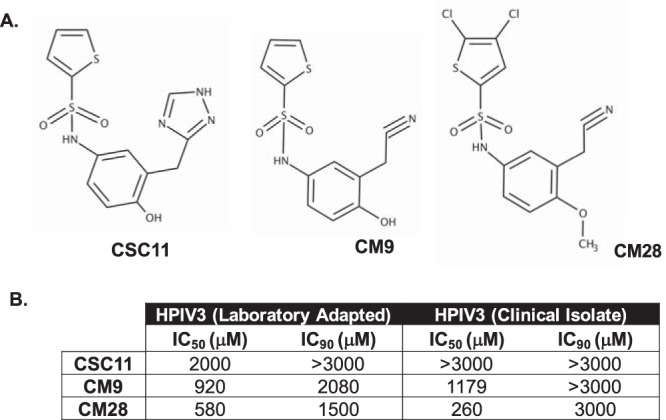
Strategy of synthesis of new inhibitors of viral entry and comparison of newly synthesized inhibitors of viral entry with CSC11. (A) Chemical structure of CSC11 compound. Each group—thiophene, hydroxyphenol, or triazole (alone or in combination)—has been modified to increase activity, resulting in structures of variations of the following CSC11 compounds shown: CM9 and CM28. (B) Comparison of properties of CSC11-derived compounds. CV1 cells grown in a monolayer culture were infected in the presence of increasing concentrations of the compounds. Viral entry was assessed by plaque reduction assay. Values corresponding to 50% inhibitory concentrations (IC_50_) and IC_90_ were then calculated for inhibition of entry by HPIV3 laboratory-adapted reference strain and HPIV3 clinical strain viruses. The values are representative of data from three experiments.

The structures of CSC11 and of two selected derivatives that were targeted in a series of chemical modifications to identify the functional groups responsible for stimulating HN to activate F are depicted in Fig. 1A. We determined whether modification of each CSC11 functional group—the thiophene R1, the hydroxyphenol R2, and the triazole R3 (see Fig. S1 in the supplemental material)—alone or in combination could preserve antiviral function and/or affect other parameters involved in the interaction between the small molecule and HN. Thirty-three molecules were synthesized and assessed for efficacy at inhibiting viral entry, as measured by plaque formation, for both laboratory-adapted strains and clinical isolate HPIV3. All 33 new compounds inhibited HPIV3 infectivity in a plaque reduction assay to various degrees, but among these, only 2 showed improved inhibitory efficacy over our prototype CSC11. The modified molecules that preserved antiviral efficacy against clinical isolate viruses were chosen for further analysis. As shown in Fig. 1B, the concentrations at which 50% and 90% inhibition of viral entry was achieved for CSC11 and the following two newly synthesized modified compounds: CM9, where the 1,2,4-triazole group on CSC11 was replaced with a nitrile group, and CM28, where the thiophene, 1,2,4-triazole, and hydroxyl groups of CSC11 were replaced with a 2,3-dichlorothiophene group, a nitrile group, and a methoxy group, respectively.

10.1128/mBio.02900-18.1 FIG S1 Structural and inhibitory properties of viral entry inhibitor library. The tables show the structure and 50% inhibitory concentrations (IC_50_) and IC_90_ of the tested compounds depicted through their respective structural R groups (R1, R2, and/or R3) and their IC_50_ and IC_90_ values as obtained through plaque reduction assay using both laboratory-adapted and clinically isolated HPIV3 strains. The values are representative of data from three experiments performed with CV1 cells infected in the presence of increasing concentrations of the listed compounds. Download FIG S1, PDF file, 0.3 MB. Copyright © 2019 Bottom-Tanzer et al. 2019 Bottom-Tanzer et al. This content is distributed under the terms of the Creative Commons Attribution 4.0 International license.

### CSC11 and CM9 do not block HN-receptor interaction for laboratory-adapted strains or clinical isolate HPIV3.

**FIG 2:**
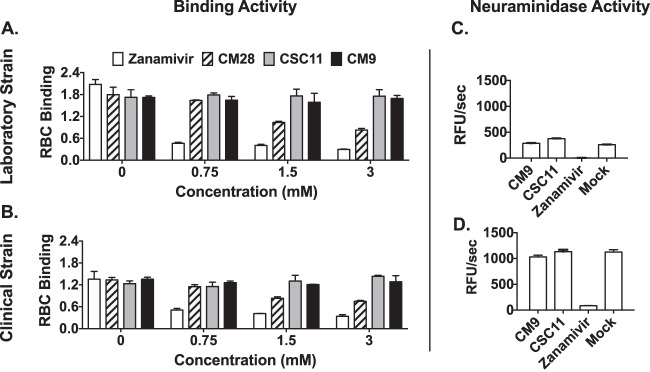
Small-molecule activity effects on HN-receptor interaction and neuraminidase activity. (A to B) Sensitivity of HN receptor binding to increasing concentrations of viral entry inhibitors (white bars, zanamivir; striped bars, CM28; gray bars, CSC11; black bars, CM9) was quantified by hemadsorption at 4°C on cells expressing HPIV3 HN from a laboratory-adapted reference strain (A) or a clinical isolate strain (B). The results are shown as absorbance at 405 nm reflecting the binding of red blood cells (RBC) (*y* axis) as a function of test compound concentration (*x* axis). Each point represents the mean of results from 3 experiments (± standard deviations [SD]), each of which was performed in triplicate. (C and D) Relative neuraminidase activity in the presence or absence of the indicated compounds (*x* axes) was assayed at 37°C and pH 5 on cell monolayers transiently expressing HN from a clinical strain (C) or a laboratory-adapted strain (D). Each bar represents results of triplicate experiments ± standard deviations; data are expressed as relative fluorescence units (RFU)/s.

We assessed the effect of the two new compounds (CM9 and CM28) compared to CSC11 on HPIV3-HN receptor binding in a hemadsorption (HAD) assay that quantitates the binding of sialic acid receptor-bearing erythrocytes (red blood cells; RBC) to HN/F coexpressing cells. For comparison, we used zanamivir (4-GU-DANA), which inhibits HN-receptor binding (13). CSC11 and CM9 (in contrast to zanamivir) did not affect receptor binding for either laboratory-adapted or clinical strain HNs (Fig. 2A and B), confirming that the inhibitory mechanism does not involve inhibition of receptor binding. In contrast, CM28 partially inhibited binding and therefore was not assessed further. We next assessed CM9 and CSC11 for their effect on neuraminidase activity. The compounds had no inhibitory effect on viral neuraminidase activity (Fig. 2C and D), indicating that CM9 does not interfere with HN’s primary receptor binding/cleaving site (site I) for either strain of HPIV3.

### CSC11 and CM9 exert a virucidal effect on clinical strain viruses.

**FIG 3:**
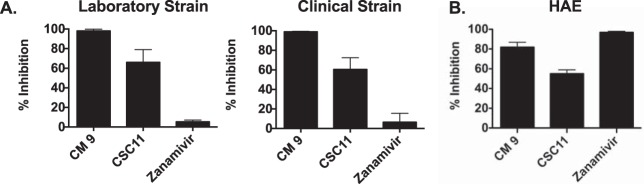
Assessment of virucidal action and *ex vivo* efficacy. (A) Aliquots of HPIV3 laboratory-adapted reference strain (left) or clinical strain (right) viral preparations were incubated with 3 mM solutions of the indicated compounds for 60 min at 37°C or left untreated. The compounds were removed using filtration columns, and the resulting viral infectivity was determined by plaque reduction assay. The effect on infectivity of the pretreatment with the compounds is shown as percent inhibition of the number of plaques compared to the number without preincubation with compounds. (C) Human airway epithelia (HAE) were infected with 4,000 PFU of the HIPV3 clinical strain in the presence of 3 mM CM9, CSC11, or zanamivir. After 90 min of incubation at 37°C, the inoculum was removed and the HAE were incubated at 37°C. Virus released from the apical surface was collected at 3 days postinfection. The percent reduction of the viral titer compared to results from untreated samples is shown on the *y* axis.

We next asked whether inhibition of viral entry is attributable to a direct and temperature-dependent virucidal effect prior to virus-target cell interaction, in line with our hypothesis that the compounds stimulate HN to trigger F at 37°C. Virions were incubated with the compounds at 37°C or 4°C for 60 min, and, after removal of the compounds, the infectivity of the treated virions was assessed by plaque reduction assay. Pretreatment of the virus with CSC11 and CM9 (but not with zanamivir) at 37°C had an irreversible effect on infectivity in both the laboratory and clinical strains (Fig. 3A). Despite removal of these compounds prior to the assay, viral entry was reduced by virtually 100% by the presence of CM9 for both viruses. Pretreatment at 4°C did not significantly inactivate either virus (data not shown), consistent with the hypothesis that these compounds affect F-triggering, which cannot occur at this temperature. To assess particle integrity, we quantitated viral RNA in infectious and noninfectious preparations as we did previously for CSC11 (7). The viral RNA levels in samples pretreated with CM9 were similar to those in samples treated with either dimethyl sulfoxide (DMSO) or zanamivir (data not shown), indicating that the decreased infectivity was due to viral inactivation and not to a loss of viral particle integrity.

### CM9 inhibits viral infection in human airway epithelium.

For clinical utility, an effective agent must limit the viral life cycle in authentic tissue. We established previously that a human airway epithelium (HAE) model closely reflects the *in vivo* behavior of a panel of HPIV3 variants (9, 14), and we employed this system to test a sialidase-based inhibitor, showing a direct correlation between the HAE and *in vivo* results (15). The HAE model was previously used to characterize the cell specificity of respiratory syncytial virus (16) and HPIV3 (17), studies that confirmed that the model replicates the paramyxovirus-HAE interactions occurring in the human lung. We assessed the effect of CM9 compared to that of CSC11 on viral infection in HAE (Fig. 3B). HAE were infected with the HPIV3 clinical isolate in the presence of CM9, CSC11, or zanamivir (which, as described above, inhibits by interfering with HN-receptor binding). After 90 min at 37°C, the inoculum was removed and the HAE cells were incubated at 37°C. The virus released from the apical surface was collected 3 days postinfection. The percentages of reduction in viral titer compared to the results seen with untreated infections are shown on the *y* axis. The viral titer in CM9-treated tissues was reduced by 80%, while the viral titer in those treated with CSC11 was reduced by approximately 55%. Zanamivir, since it blocks HN-receptor binding, is also effective via this alternative mechanism. These results support the idea of the efficacy of CM9 in the natural host and will lead to future experiments with more-potent analogs of this compound in an animal model.

### Evaluation of mechanism of action of CM9: development of conformation-specific anti-F antibodies.

**FIG 4:**
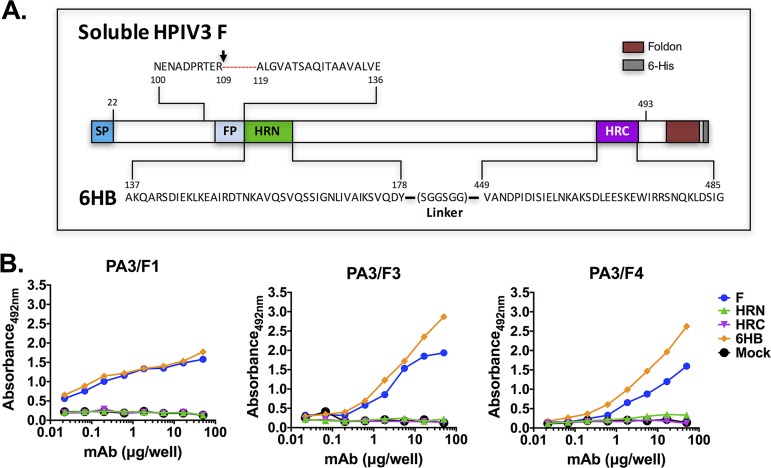
Generation and analysis of monoclonal antibodies specific for the postfusion conformation of HPIV3 F. (A) Scheme of the constructs of hPIV3 F used in this study for isolation of postfusion-specific monoclonal antibodies. The F protein scheme shows the last amino acid of its ectodomain (493), the fusion peptide (gray), the cleavage site (arrow), the C-terminal 6×His tag (dark gray), the foldon sequence (dark red), the signal peptide (blue), and the HRC and HRN domains. The sequence used for the 6HB is shown at the bottom. (B) Proteins were tested for binding in ELISA to the MAbs indicated in each panel. Results are shown as abundance at 492 nm on the *y* axis.

To directly determine whether the F protein achieves a triggered conformation as a result of compound treatment, we developed a set of antibodies to detect this state of F. Soluble F HRN and HRC regions were linked to form a protein that adopts a six-helix bundle (6HB) conformation. This structure mimics the postfusion form of the F protein that arises after the HRN and HRC regions have become associated (see Fig. 4A). Following a strategy similar to that used for human pneumovirus F proteins, the immunization with the 6HB peptide allowed the isolation of three specific monoclonal antibodies against the postfusion F conformation. This specificity was observed by antibody binding to a purified postfusion soluble HPIV3 F and to the 6HB peptide but not to either HRC or HRN alone (see Fig. 4B). Stabilization of the F construct used (see Fig. 4A) in its postfusion conformation was confirmed by electron microscopy (EM) after observation of cone-shaped molecules resembling previously described HPIV3 F trimers (see Fig. S2). Among the three antibodies isolated, the PA3/F3 demonstrated the best dose-response for both the soluble F and the 6HB.

10.1128/mBio.02900-18.2 FIG S2 (A) Gel filtration traces of the F protein depicted in Fig. 4A. The inset shows a Coomassie blue-stained SDS-PAGE gel (run under reducing conditions) representing the major peak. (B) Electron microscopy of negative-stained F protein. Some cone-shaped molecules are indicated by black arrowheads. Scale bar, 100 nm. Download FIG S2, PDF file, 4.6 MB. Copyright © 2019 Bottom-Tanzer et al. 2019 Bottom-Tanzer et al. This content is distributed under the terms of the Creative Commons Attribution 4.0 International license.

**FIG 5:**
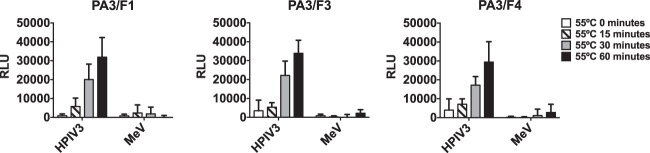
Triggering of HPIV3 F protein at 55°C: identification of the activated state of F. 293T cells expressing HPIV3 F protein (or measles F protein) were incubated overnight at 37°C. The cells were then incubated at 55°C for the indicated times and then stained with mouse MAbs recognizing the triggered conformation of HPIV3 F at 4°C. The values on the *y* axis represent the amounts of conformational antibody binding in relative luminescent units (RLU) and indicate the averages ± standard errors of the means (SEM) of results from three independent experiments performed in triplicate.

Specificity toward HPIV3-triggered F protein was further confirmed via cell-based enzyme-linked immunosorbent assay (ELISA) using heat-activated F protein (Fig. 5), adapting a cell-based ELISA that we had used previously (12). HEK 293T cells expressing either HPIV3 (clinical strain) or measles F proteins (as a control for specificity) were incubated at 55°C for the indicated times. We and others have previously shown for measles F protein that at this temperature, the thermal energy induces the F protein to adopt a posttriggered state (18, 19), making this a good control. After incubating the three monoclonal antibodies (MAbs) (1:1,000 dilution) with the cells at 4°C for 1 h, antibody binding was quantified using a β-galactosidase (β-Gal) reporter system (19). Maximal recognition of triggered HPIV3 F occurred at 60 min at 55°C; the antibodies, as expected, did not recognize measles F-expressing cells (these were detected by anti-measles F conformational antibodies; data not shown). The most sensitive of the three antibodies, PA3/F3, was used to further explore the mechanism of action of CM9 and CSC11.

### CM9 acts by causing untimely structural rearrangement of the F protein.

**FIG 6:**
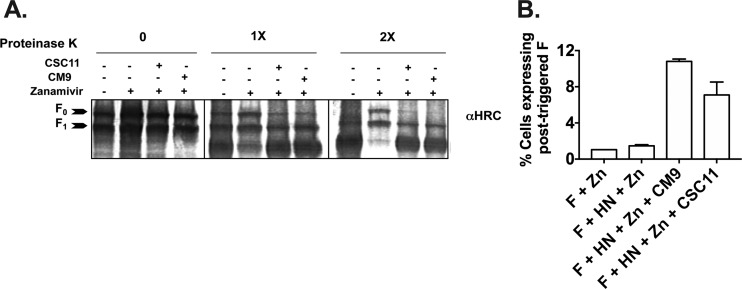
CM9 and CSC11 trigger formation of the postfusion conformation detected by limited proteolysis (A) or conformational monoclonal antibodies (B). (A) Monolayers of cells coexpressing F and HN were incubated for 60 min in medium supplemented with 3 mM CM9 or CSC11 and/or 2 mM zanamivir. The cells were lysed, and the envelope glycoproteins were immunoprecipitated and then incubated in the presence of increasing concentrations (0×, 1×, and 2×) of proteinase K. Representative Western blots show F proteolysis detected by polyclonal anti-F HRC antibodies. F0 (uncleaved) and F1 (cleaved) proteins are indicated with arrows. After receptor engagement by HN (first lane in each quadruplet) or CSC11 or CM9 treatment (third and fourth lanes in each quadruplet), F becomes susceptible to protease degradation. When receptor engagement is blocked by zanamivir and no other compound is present, F is not degraded (second lane in each quadruplet). (B) Cells cotransfected with HN and F or transfected with F only were incubated in the presence of zanamivir alone, zanamivir and CM9, or zanamivir and CSC11 and stained using PA3/F3 monoclonal antibodies. The proportions of positively stained cells were determined using Cell Profiler (∼20,000 cells were read for each condition from biological triplicates) and are shown as percentages of cells expressing posttriggered F on the *y* axis (± SD).

To determine whether CM9 causes premature activation of F, we used the antibodies developed as described above as well as a biochemical assay that we previously used to detect conformational change in F upon fusion activation (7). For the biochemical assay, we took advantage of the relative resistance of the native (prefusion state) F protein to protease K degradation. The posttriggered F is sensitive to degradation (7, 20, 21); receptor engagement by HN causes F to become sensitive to protease degradation, and conversion to protease sensitivity serves as a marker for F activation. CM9 treatment caused F to become protease sensitive (Fig. 6A), indicating that it has been activated. Cells expressing HN and F were preincubated with or without receptor engagement, and with or without CSC11 or CM9, and were then transferred to 37°C for an hour to allow fusion to occur. The HN/F complexes were immunoprecipitated and subjected to proteinase K digestion and Western blot analysis. When receptor engagement was permitted in the absence of any compound (first lane of each quadruplet), the F protein was susceptible to protease degradation and was increasingly degraded by the higher concentrations of protease. In the absence of receptor engagement (blocked by zanamivir; second lane of each quadruplet) the levels of F protein remained nearly constant, indicating that before receptor engagement, F was protected from proteinase K degradation. In the presence of CSC11 or CM9 (third and fourth lanes of each quadruplet), the F protein became susceptible to protease digestion and was degraded as fully as it had been when it was activated by HN-receptor engagement. Note that in the samples treated with CSC11 or CM9, zanamivir was present to block HN-receptor engagement (in the second lanes), indicating that it is solely the interaction with CSC11 or CM9 that accounts for the conversion to protease susceptibility. The observed conformational change in F induced by CSC11 or CM9 required the presence of the receptor-binding protein, HN.

Using the experimental conditions described for Fig. 6A, we assessed whether incubation with CSC11 or CM9 induced exposure of the postfusion epitope recognized by MAb PA3/F3. Cells cotransfected with HN and F, along with CM9 or CSC11, were incubated with zanamivir to prevent receptor engagement at 4°C and 37°C. After incubation, transfected cells were stained with a monoclonal postfusion antibody (PA3/F30; postfusion, F was quantitated using high content image analysis based on cell surface fluorescent staining. In the presence of CM9 and CSC11 at 37°C (and of zanamivir [to prevent HN-mediated F activation]), the F protein was converted to its posttriggered form. Recognition of the postfusion epitope occurred only in CM9- or CSC11-treated cells (Fig. 6B), demonstrating the ability of CM9 and CSC11 to induce the posttriggered conformation of F protein in the presence of HN, without receptor binding. We quantified the fold increase in the abundance of the posttriggered epitope compared to level seen with the same treatment at 4°C (see Table 1). Taken together, the results from these two methods—limited proteolysis and posttriggered epitope recognition—show that the small molecules promoted the posttriggered state of HPIV3 F in the absence of receptor engagement.

**TABLE 1:** Ratios between the positive cells with and without the premature-triggering compound^*a*^

Compound	Fold increase at 4^o^C	Fold increase at 37^o^C
HN +F + zn	0	0
HN +F + zn + CSC11	0.80 ± 0.42	4.97 ± 1.38
HN +F + zn + CM9	0.85 ± 0.81	7.42 ± 0.52

a *P* value for CSC11 = 0.05; *P* value for CM9 = 0.01.

## DISCUSSION

Paramyxovirus infection begins when the receptor binding protein engages the cellular receptor and, through a coordinated series of events, allows activation of the fusion protein and successful viral entry. Dysregulation of this process—whether by small-molecule antivirals (7) or by enhanced HN-F interaction—is detrimental to infectivity in the natural host. This is underscored by the differences that have recently become apparent between clinical isolate and laboratory-adapted strains of HPIV3 (14, 22, 23). Highly fusogenic laboratory-adapted virus is remarkably infectious under conditions of growth on immortalized cells and yet yields low titers when used to infect HAE cells (8, 9). However, when passaged several times on this *ex vivo* model, variants evolved with decreased efficiency of fusion activation and reduced interaction between HN and F (24), characteristic of clinical strains. Clinical strains, while they grew efficiently in both HAE and *in vivo* models, failed to grow on immortalized cells, demonstrating the profound specificity of the HN/F machinery for the authentic host (23). Even brief culture in immortalized cells provides a selection pressure that increases fusion activation from the lower activity that is required for fitness in the lung (14). Given the precision required for F activation both temporally and in terms of strength of interaction (24–26), this activation process provides an ideal target for our antiviral approach.

Targeting the fusion process of paramyxoviruses and other enveloped RNA viruses has been considered in other contexts. Several small molecules that prevent RSV F refolding have been identified, with one progressing to a clinical trial (27). Mechanistic studies suggest an association between the small molecules and the F protein, with the virus remaining infectious upon detachment of the small molecules (28). In addition to paramyxovirus activity, broadly neutralizing antibodies (29) and small molecules (30–32) against influenza virus target the conserved HA stem region in an effort to inhibit pH-induced conformational changes that mediate uncoating and virus-endosome fusion. The antiviral strategy that we propose here is conceptually different, since we searched specifically for small molecules that irreversibly inactivated the viral fusion machinery. One such compound was previously identified for influenza virus (32), and its use follows a rationale similar to that behind the proposed use of low pH (33) or aqueous arginine-based nasal sprays (34, 35) as effective influenza antivirals. Further, cellulose acetate phthalate (CAP) elicits its antiviral activity on HIV glycoprotein gp41 in a similar manner (36). Fusion inhibition is achieved with CAP by inducing the formation of the terminal 6-helix bundle conformation of gp41 and has recently been proposed in a semen-induced, intravaginally delivered anti-HIV drug (37). This suggests that premature activation of the fusion protein as an antiviral strategy can be employed not only for paramyxoviruses similar to HPIV3 but for other RNA viruses as well.

Targeting F activation in the way that we propose has an important added benefit. By activating an irreversible viral process and rendering all affected virions inert, we address the growing clinical concerns surrounding antiviral resistance. For RNA viruses in particular, a high polymerase error rate contributes to nimble evasion of antiviral activity (38–40), making the use of receptor analogs potentially an ineffective approach (41, 42). In the case of influenza antivirals, for example, rapid viral evolution has necessitated repeated development of new antiviral approaches and continued surveillance, especially in children and immunocompromised populations (43, 44).

Here, we began with CSC11, a prototype compound that we previously demonstrated pretriggers F to its postfusion conformation in the absence of receptor engagement (7). Given that it reduced infectivity by approximately 60%, we sought to increase its effectiveness by systematically modifying functional groups on the compound. After screening the resulting compounds, we selected the most potent and further tested them. We present a novel compound that results in a nearly 100% reduction of infection upon preincubation with both laboratory-adapted and clinical isolate strains of HPIV3 and an almost 40% increase in efficacy. To determine the mechanism by which these small compounds inhibit fusion, we first used a previously developed biochemical assay which exploits the relative resistance of the prefusion F state to protease K degradation and the sensitivity of the postfusion state (7, 20, 21, 45). Indeed, CSC11 and CM9 promoted protease sensitivity in the presence of HN, indicating the ability of the compounds to induce the postfusion conformation of F through interaction with the receptor binding protein. Given the complex series of conformational changes that F adopts throughout the fusion sequence, we endeavored to determine the exact F protein structure that CSC11 and CM9 induced and to refine further our understanding of the mechanism as a whole. The available experimental evidence suggested that the F protein adopted a 6HB conformation, so we developed monoclonal antibodies to fusion proteins in this conformation. When we applied these antibodies to HN/F-transfected cells incubated with CSC11, CM9, or zanamivir alone, we observed recognition of the 6HB conformation only under the compound treatment conditions. Additionally, we observed a significantly higher percentage of cells expressing postfusion F under conditions of treatment with CM9 than with CSC11, consistent with our initial observations of efficacy.

Compounds that activate viral fusion machinery in the absence of receptor binding rendering virus particles uninfectious have potentially significant clinical applications. By using our understanding of the exquisitely sensitive and specific nature of the paramyxovirus fusion process, we have developed several compounds that exert profound virucidal effects in both *in vitro* and physiologically relevant *ex vivo* models. The optimized CM9 will be assessed in a small-animal model that represents HPIV3 disease, as a counterpart to the human airway model. To further elucidate the mechanism of action of these compounds, we developed novel monoclonal antibodies specific for the postfusion conformation of F. Not only did they confirm our hypothesis but they also will be invaluable for future structural studies of HPIV3 F, a protein against which a historically limited repertoire of antibodies has been available. Now that we have validated these antibodies as being specific for the posttriggered HPIV3 F, they can be used in a wide range of settings to confirm F activation and can be applied in high-throughput assays to screen for activated F. Together, these advances provide not only the foundation for a new antiviral strategy toward RNA viruses but also the means to further refine future studies.

## MATERIALS AND METHODS

### Cells and viruses.

293T (human kidney epithelial) and CV-1 (African green monkey kidney) cells were grown in Dulbecco’s modification of Eagle’s medium (DMEM; Thermo Fisher) supplemented with 10% fetal bovine serum and antibiotics. Stocks of laboratory-adapted HPIV3 were made in CV-1 cells from virus that was subjected to plaque purification four times. Virus was collected 36 to 48 h postinfection and stored at −80°C.

Stocks of clinical strain viruses were prepared in HAE cultures. HAE cultures were infected by applying 200 µl of EpiAirway growth medium containing 4,000 PFU of the HPIV3 clinical isolate to the apical surface for 90 min at 37°C. Following incubation, the medium containing the inoculum was removed, and cultures were grown at 37°C and fed each day with 1 ml of medium basolaterally. Three to 5 days postinfection, viruses were harvested by adding 200 µl of Opti-MEM (Thermo Fisher) apically for 30 min at 37°C. The suspension was then collected, and viral titers were determined via plaque assay as described previously (9).

### HAE cultures.

The EpiAirway AIR-100 tissue model (MatTek Corporation) consists of normal human-derived tracheobronchial epithelial cells that have been cultured to form a pseudostratified, highly differentiated mucociliary epithelium closely resembling that of airway epithelial tissue *in vivo*. Upon receipt from the manufacturer, HAE cultures were transferred into six-well plates (containing 1.0 ml medium per well), with the apical surface remaining exposed to air and incubated at 37°C in 5% CO_2_ overnight, as previously described (9).

### Infection and treatment of HAE cells and measurement of viral titers from infected HAE cells.

HAE cultures were infected by applying 200 µl of EpiAirway medium containing 4,000 PFU of laboratory-adapted or clinical strain HPIV3. Viral suspensions were applied to the apical surface of HAE tissues for a 90-minute adsorption period at 37°C in the presence or absence of 3 mM CSC11, CM9, or zanamivir. At 90 min, the medium containing the inoculum was removed, and cultures were placed at 37°C and fed each day with 1.0 ml medium via the basolateral surface. Viruses were harvested by adding 200 µl Opti-MEM per well to the apical surface of HAE cultures and allowed to equilibrate for 30 min at 37°C. The suspension was then collected, and viral titers were determined as previously described (9).

### Chemicals.

Zanamivir (4-GU-DANA) was prepared from Relenza Rotadisks (5 mg zanamivir with lactose). A 50 mM stock solution was prepared by dissolving each 5-mg blister capsule in 285 µl Optimem (Gibco). Alternatively, zanamivir (Acme Biosciences) was dissolved in Opti-MEM to reach a concentration of 50 mM and stored at −80°C. CSC compound derivatives were synthesized by Charnwood Molecular Ltd. Stock solutions (100 mM) were prepared in DMSO. Stock solutions were stored at −20°C.

### Transient expression of HPIV3 HN and F constructs.

HPIV3 HN and F genes from laboratory strains and clinical isolate viruses were cloned into pCAGGS as previously described (22). The constructs used in the assay for F protein triggering, HN-N-Venus and F-C-CFP, were prepared as previously described (10). Transfections were performed with Lipofectamine 2000 (Invitrogen), according to the manufacturer’s instructions.

### Plaque reduction assay.

The effect of the compounds on plaque number was assessed as described previously (7, 13, 46). Briefly, CV-1 cell monolayers were infected with 100 PFU of the indicated virus in the presence of serial dilutions of the indicated compounds. After 90 min, the plates were overlaid with either agarose or methylcellulose; At 24 h later, the agarose was removed, cells were fixed and immunostained, and the plaques (or single infected cells for the clinical strain) were counted.

### Hemadsorption assays.

Hemadsorption (HAD) was performed and quantified as previously described (47). Briefly, growth medium from HN-transfected 293T cell monolayers in 48-well Biocoat plates (Becton Dickinson Labware) was aspirated and replaced with 150 µl of CO_2_-independent medium (pH 7.3; Gibco) containing different concentrations of compounds and 1% RBC in serum-free CO_2_-independent medium and placed at 4°C for 30 min. The wells were then washed three times with 150 µl cold CO_2_-independent medium. The bound RBCs were lysed with 200 µl RBC lysis solution (0.145 M NH_4_Cl, 17 mM Tris HCl), and absorbance was read at 405 nm using a Spectramax M5 (Molecular Devices) microplate reader.

### Measurement of neuraminidase activity.

Assays were performed in transiently transfected 293T cell monolayers, as described previously (7, 47, 48). Briefly, 293T cells expressing either laboratory-adapted or clinical strain HN were added to 96-well plates in CO_2_-independent medium at pH 5.0. After addition of reaction mixtures containing 20 mM 2’-(4-methylumbelliferyl)-alpha-d-N-acetylneuraminic acid (Toronto Research Chemicals Inc.) substrate and different concentrations of CSC compounds, the plates were incubated at 37°C for 1 h. Throughout this period, fluorescence resulting from hydrolysis of the substrate was read at a 365-nm excitation wavelength and a 450-nm emission wavelength using a Spectramax M5 microplate reader.

### Virucidal assay and real-time (RT)-quantitative PCR analysis.

Aliquots of HPIV3 viral preparations were incubated for 1 h at 37°C in Opti-MEM supplemented with the indicated compounds or DMSO. After incubation, the samples were diluted with Opti-MEM and cleared of compounds using Ultrafree MC filters (Millipore). The viral particles were then collected from the filters in Opti-MEM and their infectivity was determined by plaque reduction assay. Using a one-step real-time quantitative PCR HPIV3 detection kit (Primer Design Ltd.) and a Realplex2 Mastercycler (Eppendorf), the total amount of viral RNA in each sample was determined (7).

### Biochemical detection of fusion protein triggering.

293T cell monolayers, transiently transfected with HN-N-Venus and F-C-CFP, or with F-C-CFP alone, were incubated overnight in DMEM supplemented with 3 mM zanamivir to prevent fusion. The transfected cells were washed with Opti-MEM and incubated for 1 h at 37°C in Opti-MEM supplemented with 100 μg/ml cycloheximide (to prevent *de novo* protein synthesis) only or additionally supplemented with 2 mM zanamivir or 2 mM zanamivir and 3 mM compound. The cells were then lysed in DH buffer (50 mM HEPES, 100 mM NaCl, 0.005 g/ml dodecyl maltoside, complete protease inhibitor cocktail [Roche]). HN and F were immunoprecipitated from postnuclear lysates with anti-green fluorescent protein (anti-GFP) antibody-conjugated agarose beads (Santa Cruz) for 2 h at 4°C, washed, and resuspended in phosphate-buffered saline (PBS) and then incubated in the absence or presence of proteinase K (Sigma) (0.64 × 10^−5 ^mg/ml [1×] or 1.28 × 10^−5 ^mg/ml [2×]) for up to 90 min. Complete protease inhibitor cocktail (Roche) was added to stop proteinase K digestion after 90 min. The protein-bead complexes were supplemented with Laemmli sample buffer, heated to 99°C for 5 min, and then subjected to SDS-PAGE and Western blot analysis with polyclonal antibodies to the HRC region of F. Relative protein levels were quantified using a Kodak ImageStation and Kodak Molecular Imaging software (7, 20, 21).

### Protein expression and purification.

The HPIV3 heptad repeat (HR) domains at the N and C termini (HRN and HRC) and the 6 helix bundle complex (6HB) were produced as previously reported (49). Briefly, the N42-SGG (AKQARSDIEKLKEAIRDTNKAVQSVQSSIGNLIVAIKSVQDYSGG) and C37-SGG (SGGVANDPIDISIELNKAKSDLEESKEWIRRSNQKLDSIG) fragments were subcloned into the pTMHa expression vector encoding a N-terminal His_9_-TrpLE chimeric protein (50). The chimeric proteins were expressed in Escherichia coli strain BL21(DE3)/pLysS (Novagen) and purified from insoluble bacterial inclusion bodies by nickel-nitrilotriacetic acid (Ni-NTA) metal-affinity chromatography following published procedures (50). The His_9_-TrpLE leader sequence was subsequently cleaved off by using cyanogen bromide (CNBr). The N42(L6)C37 construct was expressed in E. coli by using a modified pET3a vector (Novagen). Cells were lysed by glacial acetic acid and centrifuged to separate the soluble fraction from inclusion bodies. The soluble fraction containing protein was subsequently dialyzed into 5% acetic acid overnight at 4°C. All proteins were purified to homogeneity by reverse-phase high-performance liquid chromatography (HPLC) (Waters, Inc.) on a Vydac C_18_ preparative column (Hesperia, CA) using a water-acetonitrile gradient in the presence of 0.1% trifluoroacetic acid and lyophilized. Protein identities were confirmed by electrospray mass spectrometry (PerSeptive Biosystems Voyager Elite, Cambridge, MA). For expression of soluble HPIV3 postfusion F protein, a pCAGGs plasmid encoding the ectodomain (amino acids 1 to 493) from strain CI-1 was prepared by Epoch Biolabs. To avoid protein aggregation in the case of efficient F cleavage, this construct also contained a deletion of the first nine residues of the fusion peptide (amino acids 110 to 118). The foldon trimerization domain was also added at the C terminus of the F ectodomain flanked upstream by a tobacco etch virus (TEV) protease site and downstream by a Xa protease site, followed by a 6×His tag. Hence, the complete amino acid sequence of the C-terminal was as follows: *SGRENLYFQG*GGG**GSGYIPEAPRDQAYVRKDGEWVLLSTFL**GG*TEGR*HHHHHH (TEV and Xa sequences are in italics and underlined, and foldon sequences are in boldface). Other sequences correspond to linkers and histidines. Plasmid was used to transfect FreeStyle 293-F cells (Invitrogen), and protein F was purified from media after immobilized metal affinity chromatography (Ni2+ column) followed by two rounds of size exclusion chromatography as previously described (51). The purity and integrity of the purified protein were analyzed by SDS-PAGE and Coomassie blue staining under reducing conditions.

### Monoclonal antibodies that recognize the postfusion F.

Female BALB/c mice were inoculated intramuscularly (i.m.) and boosted 4 weeks later with 6HB peptide (20 µg for each inoculation). Four days after the last inoculation, mice were sacrificed by isoflurane (ISOVA) inhalation and their splenocytes fused to Sp2-0 myeloma cells with polyethylene glycol 4000 (PEG 4000). Hybridomas were selected in ClonaCell-HY Liquid HAT Selection Medium E (StemCell Technologies). Postfusion-specific antibody production in hybridoma supernatants was tested by ELISA using the soluble HPIV3 F protein as described below. Positive cultures were recloned at least twice by limiting dilution using ClonaCell-HY Medium E (StemCell Technologies). The postfusion specificity of supernatant antibodies after cloning was confirmed by ELISA using the soluble HPIV3 F protein and the HRC HRN and 6HB peptides.

Hybridoma cells were maintained in ClonaCell-HY Medium E (StemCell Technologies). For antibody production, flasks were seeded at a confluence of 1 × 10^6^ cells/ml in hybridoma serum-free medium (Gibco). To harvest antibodies, cells and medium were centrifuged at 4°C for 10 min at 1,000 relative centrifugal force (rcf). Supernatant fluid containing antibodies was collected with a 0.22-µm-pore-size filter (Millipore) and stored at −20°C or at 4°C for a maximum of 2 weeks.

### Enzyme-linked immunosorbent assay (ELISA).

Wells of 96-well microtiter plates were coated with 40 ng/well of purified protein or peptides using PBS buffer for 16 h at 4°C. Nonspecific binding was blocked with 2% porcine serum–PBS–0.05% Tween. Serial volumes of antibody supernatants were then added to the wells for 1 h at room temperature, followed sequentially by an excess of anti-mouse IgG horseradish peroxidase-linked antibody (GE-Healthcare) and substrate (OPD [o-phenylenediamine dihydrochloride]; Sigma). Extensive washing with water followed each step. Optical density was read at 492 nm.

### Ethics statement.

Animal studies were performed under the regulations of Spanish and European legislation concerning vivisection and the use of genetically modified organisms. Protocols were approved by the Comité de Ética de la Investigación y del Bienestar Animal of Instituto de Salud Carlos III (CBA PA 19_2012).

### Electron microscopy.

Soluble HPIV3 postfusion F was applied to glow-discharged carbon-coated grids and negatively stained with 1% uranyl formate. Images were recorded on a Gatan ES1000W charge-coupled-device (CCD) camera in a JEOL JEM-1011 microscope operated at 100 kV.

### β-Gal assay for detection of fusion protein triggering with F-conformation-specific MAbs.

293T cells transiently transfected with viral glycoprotein constructs were incubated overnight at 37°C in complete medium (DMEM, 10% fetal bovine serum [FBS]). At 20 h posttransfection, cells were transferred to conditions corresponding to the temperatures and times indicated in the figure. Thereafter, cells were incubated with mouse monoclonal antibodies (MAbs) that specifically detect HPIV3 F in its posttriggered conformation (1:1,000) for 1 h on ice. Cells were washed with PBS and then incubated for 1 h on ice with biotin-conjugated anti-mouse secondary antibody (Life Technologies) (1:500). Cells were washed with PBS and then fixed for 10 min on ice with 4% paraformaldehyde (PFA). Cells were then washed twice with PBS, blocked for 20 min on ice with 3% bovine serum albumin (BSA)–PBS, washed, and then incubated for 1 h on ice with streptavidin conjugated with β-Gal (Life Technologies) (1:1,000). Cells were washed with PBS, the β-galactosidase substrate (Applied Biosystems) (1:50) was added, and luminescence was measured by the use of a Infinite M1000PRO (Tecan) microplate reader.

### Detection and quantitation of triggered F.

Ninety-six-well plates were seeded with 293T cells. The following day, cells were transfected with combinations of HPIV3 laboratory-adapted F and HN and Lipofectamine 2000. After 3 h, complete medium with 2 mM zanamivir was added to the cells and they were incubated overnight at 37°C. The next day, cells were brought to 4°C, washed, and overlaid with cycloheximide and 2 mM zanamivir or with cycloheximide, 2 mM zanamivir, and 3 mM CM9 or CSC11. The plates were then either left at 4°C or maintained at 37°C for 60 min. Following treatment, plates were put on ice and incubated with purified PA3/F3 antibody (10 µg/ml) for 60 min, washed with Dulbecco's phosphate-buffered saline (DPBS), fixed with 4% PFA for 15 min, blocked with 3% BSA, incubated in the dark for 60 min with 1:500 anti-mouse Alexa Fluor 555 (Thermo Fisher)–3% BSA, and then incubated in a 1:1,000 dilution of DAPI (4′,6-diamidino-2-phenylindole; Thermo Fisher) for 60 min. Plates were washed, 0.01% sodium azide was added, and plates were imaged via the use of an IN Cell Analyzer. Percentages of positively recognized cells were determined using Cell Profiler.
