# Supplementary material for: Inhibiting Human Parainfluenza Virus Infection by Preactivating the Cell Entry Mechanism
Source: mBio. 2019 Feb 19;10(1):e02900-18. doi: 10.1128/mBio.02900-18 (PMC6381285; doi:10.1128/mBio.02900-18)
Supplement: FIG S1 [file mBio.02900-18-sf001.pdf]

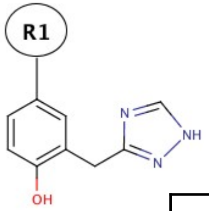

S1A

		HPIV3 (Laboratory Adapted)		HPIV3 (Clinical Isolate)	
**R1 Group**	**Name**	**IC50 (μM)**	**IC90 (μM)**	**IC50 (μM)**	**IC90 (μM)**
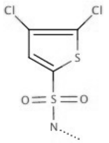	**CM1**	2260	> 3000	> 3000	> 3000
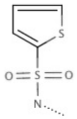	**CM2**	> 3000	> 3000	> 3000	> 3000
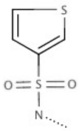	**CM3**	> 3000	> 3000	> 3000	> 3000
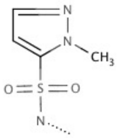	**CM8**	> 3000	> 3000	> 3000	> 3000
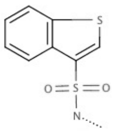	**CM11**	2020	> 3000	2800	> 3000
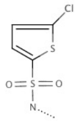	**CM12**	2740	> 3000	> 3000	> 3000
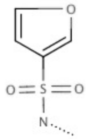	**CM14**	2120	> 3000	1500	> 3000

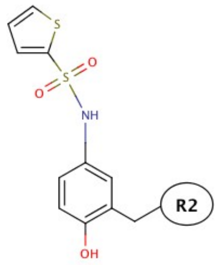

S1B

R2 Group	Name	HPIV3 (Laboratory Adapted) IC50 (μM)	HPIV3 (Laboratory Adapted) IC90 (μM)	HPIV3 (Clinical Isolate) IC50 (μM)	HPIV3 (Clinical Isolate) IC90 (μM)
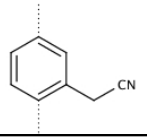	**CM9**	920	2080	1179	> 3000
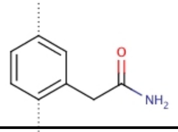	**CM4**	1900	> 3000		
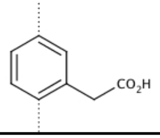	**CM121**	> 3000	> 3000	> 3000	> 3000
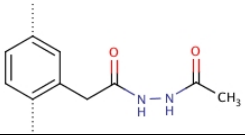	**CM8A**	> 3000	> 3000	> 3000	> 3000
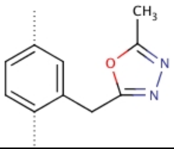	**CM141**	> 3000	> 3000	> 3000	> 3000

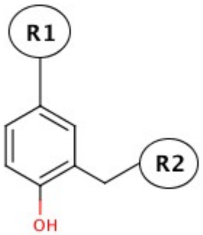

S1C

			HPIV3 (Laboratory Adapted)		HPIV3 (Clinical Isolate)	
R1 Group	R2 Group	Name	IC50 (μM)	IC90 (μM)	IC50 (μM)	IC90 (μM)
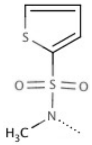	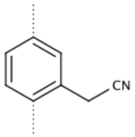	**CM19**	1140	1460	1225	> 3000
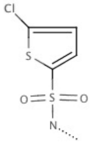	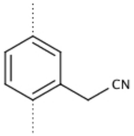	**CM29**	960	> 3000	1640	> 3000
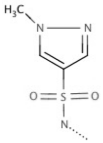	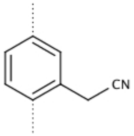	**CM34**	2220	> 3000	> 3000	> 3000
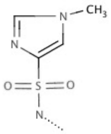	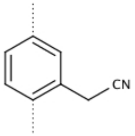	**CM35**	2100	> 3000		
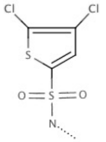	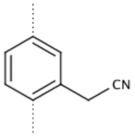	**CM36**	1100	> 3000	2180	> 3000

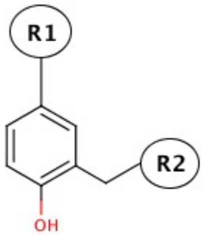

S1D

			HPIV3 (Laboratory Adapted)		HPIV3 (Clinical Isolate)	
**R1 Group**	**R2 Group**	**Name**	**IC50 (μM)**	**IC90 (μM)**	**IC50 (μM)**	**IC90 (μM)**
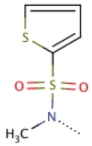	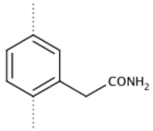	**CM19A**	> 3000	> 3000	> 3000	> 3000
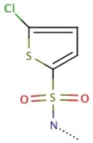	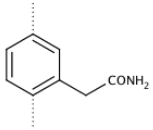	**CM30**	> 3000	> 3000	> 3000	> 3000
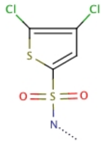	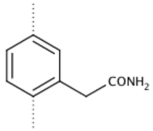	**CM33**	1140	2540	1360	>3000
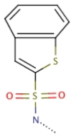	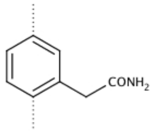	**CM42**	2250	> 3000	2380	> 3000
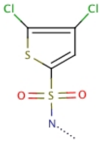	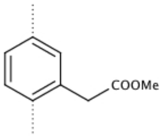	**CM37**	750	1360		

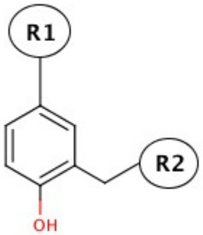

S1E

			HPIV3 (Laboratory Adapted)		HPIV3 (Clinical Isolate)	
R1 Group	R2 Group	Name	IC50 (μM)	IC90 (μM)	IC50 (μM)	IC90 (μM)
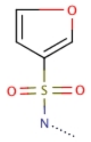	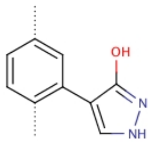	**CM27**	2220	>3000		
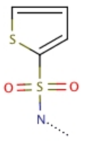	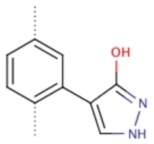	**CM31**	> 3000	> 3000	> 3000	> 3000
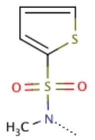	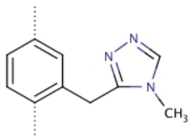	**CM39**	> 3000	> 3000	> 3000	> 3000
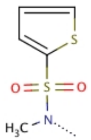	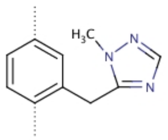	**CM40**	> 3000	> 3000	2840	> 3000
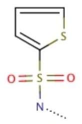	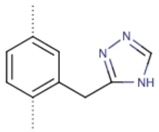	**CM44**	> 3000	> 3000	> 3000	> 3000

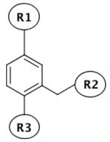

S1F

				HPIV3 (Laboratory Adapted)		HPIV3 (Clinical Isolate)	
**R1 Group**	**R2 Group**	**R3 Group**	**Name**	**IC50 (μM)**	**IC90 (μM)**	**IC50 (μM)**	**IC90 (μM)**
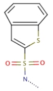	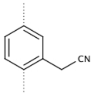	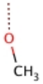	**CM22**	540	970	660	1550
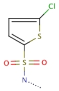	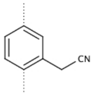	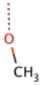	**CM21**	2440	>3000	975	2350
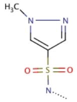	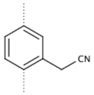	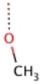	**CM24**	>3000	> 3000	>3000	>3000
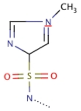	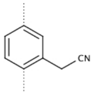	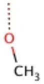	**CM25**	>3000	> 3000	>3000	> 3000
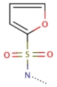	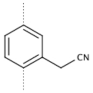	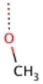	**CM26**	>3000	> 3000	>3000	> 3000
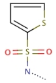	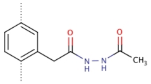	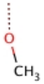	**CM81**	>3000	> 3000	>3000	> 3000
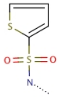	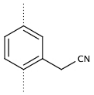	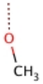	**CM163**	1460	2110	275	2875
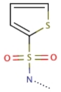	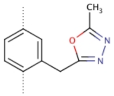	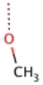	**CM6**	>3000	> 3000	>3000	> 3000
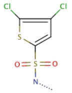	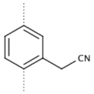	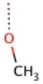	**CM28**	580	1500	260	3000
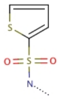	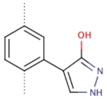	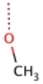	**CM32**	> 3000	> 3000		
