# Supplementary figures and images for: Inhibiting Human Parainfluenza Virus Infection by Preactivating the Cell Entry Mechanism
Source: mBio. 2019 Feb 19;10(1):e02900-18. doi: 10.1128/mBio.02900-18 (PMC6381285; doi:10.1128/mBio.02900-18)

A.

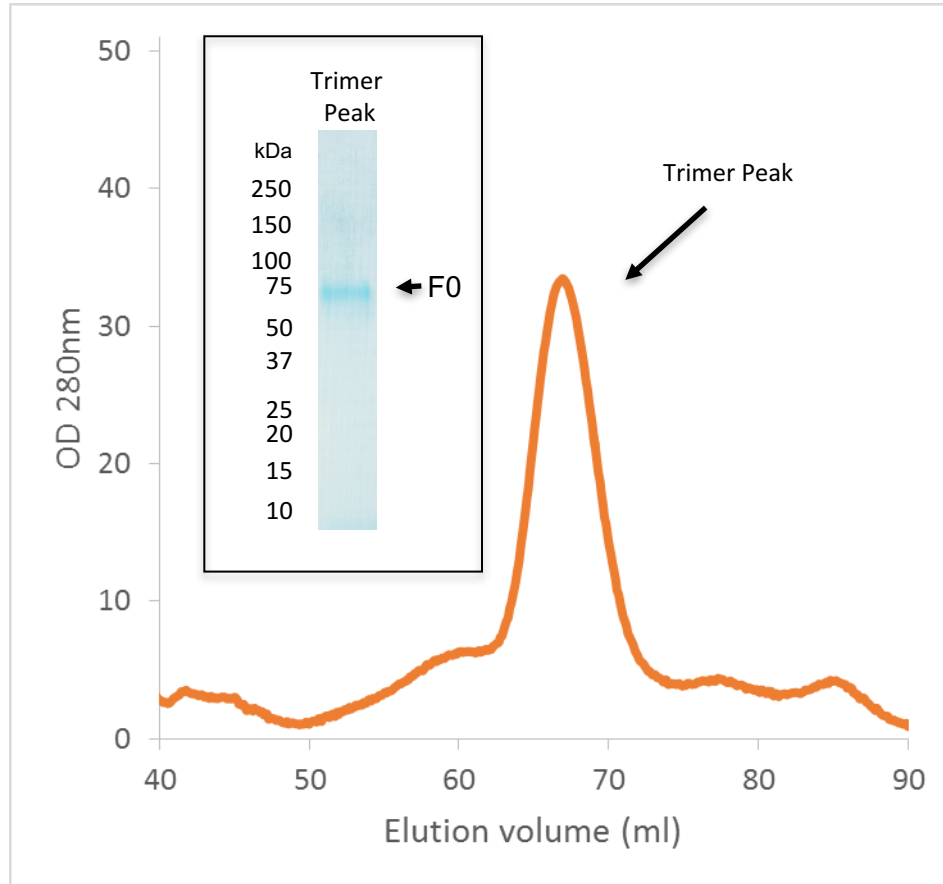

B.

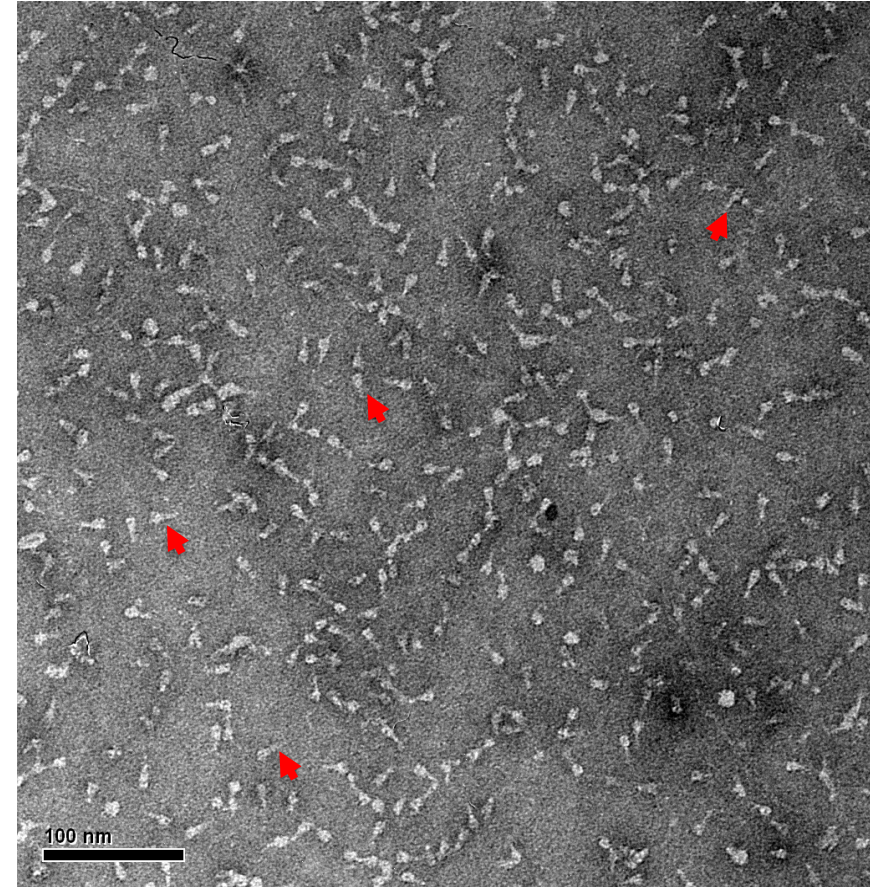

Supplement: FIG S2 [file mBio.02900-18-sf002.pdf]
